# A Retrospective Analysis of the Effects of Concomitant Use of Intra-Aortic Balloon Pump (IABP) and Veno-Arterial Extracorporeal Membrane Oxygenation (va-ECMO) Therapy on Procedural Brain Infarction

**DOI:** 10.3390/diagnostics15060699

**Published:** 2025-03-12

**Authors:** Farid Ziayee, Hannan Dalyanoglu, Christian Schnitzler, Kai Jannusch, Matthias Boschheidgen, Judith Boeven, Hug Aubin, Bernd Turowski, Marius Georg Kaschner, Christian Mathys

**Affiliations:** 1 Department of Diagnostic and Interventional Radiology, Medical Faculty, University Dusseldorf, 40225 Dusseldorf, Germany; farid.ziayee@med.uni-duesseldorf.de (F.Z.); christian.schnitzler@med.uni-duesseldorf.de (C.S.); kai.jannusch@med.uni-duesseldorf.de (K.J.);; 2 Department of Cardiovascular Surgery, Medical Faculty, University Dusseldorf, 40225 Dusseldorf, Germany; 3 Institute of Radiology and Neuroradiology, Evangelisches Krankenhaus Oldenburg, Universitätsmedizin Oldenburg, Steinweg 13–17, 26122 Oldenburg, Germany

**Keywords:** thromboembolism, watershed, stroke, intra-aortic balloon pump, extracorporeal membrane oxygenation

## Abstract

**Background/Objectives:** Brain ischemia is a frequent complication in patients undergoing veno-arterial extracorporeal membrane oxygenation (va-ECMO) therapy due to hypoperfusion, low oxygenation, and thromboembolism. While concomitant intra-aortic balloon pump (IABP) therapy may improve the perfusion of the supra-aortic branches, it may also favor thromboembolism. This retrospective study aimed to evaluate the effects of combined va-ECMO and IABP therapy on procedural brain infarction compared to va-ECMO therapy alone, with a specific focus on analyzing the types of infarctions. **Methods:** Cranial computed tomography (CCT) scans of consecutive patients receiving va-ECMO therapy were analyzed retrospectively. Subgroups were formed for patients with combined therapy (ECMO and IABP) and va-ECMO therapy only. The types of infarctions and the potential impacts of va-ECMO vs. combined therapy with IABP on stroke were investigated. **Results:** Overall, 146 patients (36 female, 110 male, mean age 61 ± 13.3 years) were included, with 69 undergoing combined therapy and 77 patients receiving va-ECMO therapy alone. In total, 14 stroke events occurred in 11 patients in the ECMO-only group and there were 12 events in 12 patients in the ECMO + IABP-group, showing no significant difference (*p* = 0.61). The majority of infarctions were of thromboembolic (*n* = 23; 88%) origin, with 14 stroke-events in 12 patients in the ECMO + IABP-group and 9 stroke events in the ECMO-only group. The survival rate within 30 days of treatment was 29% in the ECMO-only group and 32% in the ECMO + IABP group. **Conclusions:** The results of this retrospective study show that concomitant IABP therapy appears to be neither protective nor more hazardous in relation to ECMO-related stroke. Thus, the indication for additional IABP therapy should be assessed independently from the procedural risk of brain ischemia. Thromboembolic infarctions seem to represent the most common type of infarction in ECMO, especially within the first 48 h of treatment.

## 1. Introduction

Extracorporeal membrane oxygenation (ECMO) has emerged as a pivotal component of modern extracorporeal life support, serving as a vital temporary measure for patients requiring cardiopulmonary support in critical situations [1]. It provides a crucial bridge to more definitive interventions, such as heart transplantation or recovery from severe organ failure [2]. Despite its life-saving potential, ECMO therapy is associated with a range of severe and often life-threatening complications that can significantly impact patient outcomes.

In the case of veno-arterial ECMO (va-ECMO), venous blood is extracted from the inferior vena cava and returned to the arterial system. This technique is predominantly employed as a bridge therapy, aiming to stabilize patients with acute cardiogenic shock or severe cardiac failure. However, va-ECMO carries considerable risks, including the potential for brain ischemia, which can result in devastating neurological outcomes [3,4].

Despite significant advancements in ECMO management over the past four decades, serious complications remain common, including bleeding, vascular injury, and ischemia affecting the brain, visceral organs, and limbs. Diagnosing brain ischemia in ECMO patients poses unique challenges due to their often altered consciousness and complex clinical presentations, which can lead to the potential underreporting of neurological complications [5,6]. Current estimates of neurological complications among ECMO patients vary widely, ranging from 1–78% [7]. The most prevalent cerebral complications associated with ECMO therapy include intracranial hemorrhage, stroke, and intracranial edema [5,6,7].

Brain infarction during va-ECMO can arise from several mechanisms. One potential cause is the formation of a thrombus within the ECMO system that may enter the cerebral circulation through either central or peripheral cannulation. This can result in territorial or small lacunar infarctions. Hemodynamic alterations, especially those associated with peripheral va-ECMO, can reduce blood flow in supraaortic vessels, resulting in hemodynamic infarctions. Furthermore, a watershed phenomenon in the aortic arc and device dysfunction may lead to hepoxemic infarctions in the basal ganglia or cortex [6,7,8,9].

The intra-aortic balloon pump (IABP) has been introduced as an adjunctive therapy to enhance the efficacy of peripheral va-ECMO. The IABP operates by inflating during the diastole and deflating during the systole, which enhances diastolic pressure and is thought to reduce afterload. This mechanism can improve cardiac output by up to 20% and enhance perfusion to the coronary arteries, supra-aortic branches, and visceral vessels [10]. However, the combined use of IABP with va-ECMO remains a topic of debate among clinicians [3,11]. Despite its potential cerebral benefits, the IABP is a thrombogenic device, raising concerns about the risk of thromboembolic complications.

Currently, there is a notable gap in the literature regarding the incidence of brain infarctions in patients receiving combined ECMO and IABP therapy. The simultaneous implantation of IABP during peripheral va-ECMO is not considered standard practice, and its effect on stroke risk remains largely unclear. We hypothesize that the addition of IABP therapy may reduce the risk of hemodynamic intracranial infarctions by improving cerebral perfusion [12]. Conversely, an embolus originating from the ECMO system during peripheral cannulation may be more likely to reach the cerebral circulation in the presence of IABP therapy. Therefore, unless the watershed area is distal to the supra-aortic vessels, the use of IABP may inadvertently increase the risk of thromboembolic infarctions in this scenario. To our knowledge, no studies have specifically reported the incidence rates of brain infarctions or differentiated between the types of infarctions (e.g., hypoxic, thromboembolic, or hemodynamic) in patients undergoing combined va-ECMO and IABP therapy.

The primary objective of this study is to assess the impact of adjunctive IABP therapy on the risk of brain ischemia in patients receiving va-ECMO therapy and to classify the types of infarctions observed. By enhancing our understanding of these relationships, we aim to improve patient management strategies and clinical outcomes in this vulnerable population.

## 2. Materials and Methods

This retrospective study analyzed data from consecutive patients who underwent va-ECMO therapy between January 2010 and December 2015 at the Department of Cardiovascular Surgery, University Hospital of Düsseldorf, Germany. This study, approved by the local institutional review board, aimed to assess the impact of va-ECMO with or without adjunct intra-aortic balloon pump (IABP) therapy on the occurrence of brain infarctions. Patients were divided into two groups based on their treatment modality: the ECMO-only group and the ECMO + IABP group. The choice to employ va-ECMO alone or alongside IABP for patients in cardiogenic shock depends on a range of clinical parameters and individual patient conditions. Va-ECMO is often preferred initially for its robust circulatory and respiratory support capabilities. The addition of IABP was performed in cases of severe left ventricular dysfunction or when afterload reduction was essential to optimize left ventricular unloading and enhance coronary perfusion. In instances where va-ECMO alone led to increased afterload and inadequate left ventricular unloading, IABP was integrated to mitigate complications such as pulmonary edema and left ventricular thrombus formation.

### 2.1. Inclusion and Exclusion Criteria

#### 2.1.1. Inclusion Criteria Included the Following

Patients treated with va-ECMO.

A minimum age of 18 years.

Cranial computed tomography (CCT) performed during ECMO therapy.

#### 2.1.2. Exclusion Criteria Included the Following

Patients who received additional left or right ventricular assist devices (LVADs/RVADs).

The incorrect positioning of the IABP tip or any ECMO component.

Patients with hypoxic infarctions not associated with ECMO therapy.

The proper placement of the IABP tip was defined as being distal to the left subclavian artery within the thoracic aorta. For peripheral cannulation, the arterial ECMO cannula was inserted into either the external or common iliac artery. For central cannulation, it was positioned in the ascending aorta. The venous cannula was inserted into the inferior vena cava, right atrium, or superior vena cava. Cannulation positions and IABP placements were verified through thoracic X-ray analysis and CT imaging.

#### 2.1.3. Data Collection

Patient baseline characteristics were gathered, including details on cannulation sites, the duration of ECMO therapy, indications for device therapy, occurrences of cardiopulmonary resuscitation (CPR), 30-day survival rates post-device implantation, and neuron-specific enolase (NSE) levels. This information was extracted from the hospital’s electronic health records and digital medical information system.

#### 2.1.4. Imaging Analysis

Cranial computed tomography (CCT) scans were performed using four different CT systems from Siemens Healthcare GmbH (Erlangen, Germany). All CCT scans followed a standardized protocol with supine patient positioning to ensure consistency across images. Detailed parameters for each CT system are outlined in Table 1.

**Figure 1:**
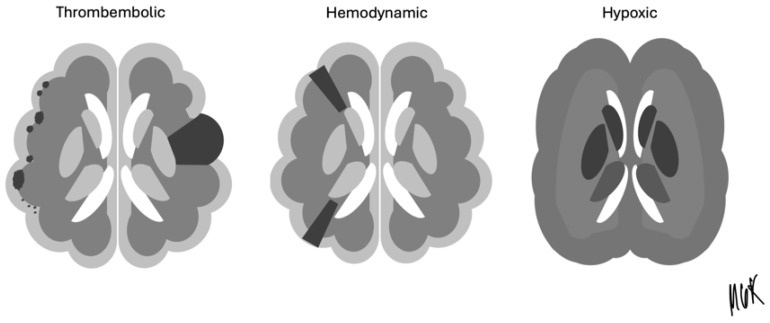
Thromboembolic, hemodynamic, and hypoxic stroke pattern for the classification of infarction.

The CCT images were retrospectively reviewed by a team of board-certified radiologists and neuroradiologists, working based on consensus to ensure the accuracy of findings. Brain infarctions were identified and classified based on lesion age, using established radiological criteria to differentiate lesions as acute, subacute, or chronic. This classification enabled the detailed analysis of the temporal relationship between infarctions and ECMO therapy. All CCTs during device therapy were included, so that, eventually, early infarctions missed on the first CCT could potentially be detected in the follow-up scan. Ischemic strokes in the ECMO and IABP group were only taken into account after the implantation of both devices. Each infarction was further categorized as a thromboembolic, hemodynamic, or hypoxemic type based on established criteria from previous studies (Figure 1). Infarctions were assigned to vascular territories: anterior, medial and posterior cerebral arteries (ACAs, MCAs, and PCAs), superior, posterior inferior and anterior inferior cerebellar arteries (SCAs, PICAs, and AICAs), and basilar arteries (BAs). Infarctions were categorized as thromboembolic, hemodynamic, or global cerebral hypoxemia.

The Alberta Stroke Program Early CT Score (ASPECTS) was used to quantify parenchymal involvement in the MCA territory of each side [13]. A score of 10 points represents healthy MCA territory, with one point deducted for each affected region (the nucleus caudatus, putamen, capsula interna, insular cortex, frontal operculum, anterior temporal lobe, and posterior temporal lobe, as well as the cortex regions immediately superior to the frontal operculum and the anterior and posterior lobe). A modified ASPECTS was introduced for other territories, with a maximum score of 19 points per side, accounting for ACA, PCA, cerebellar arteries, and BA territories. In addition to the ASPECTS, 2 more points were deducted for ACA territory (1 point for less than and 1 point for more than 50% of the territory), 3 points were deducted for PCA territory (1 point for the medial temporal lobe, 1 point for the thalamus, and 1 point for the occipital lobe), 1 point for SCA, AICA, and PICA each and 1 point for BA. Patients with multiple stroke events had each event recorded and classified to provide a comprehensive view of complications.

#### 2.1.5. Statistical Analysis

Statistical analysis was carried out using SPSS Statistics 24 software (IBM, Armonk, NY, USA). The chi-square test was applied to dichotomous data with a sample size of five or greater. In cases with smaller sample sizes, Fisher’s exact test was used to ensure precision. The Kolmogorov–Smirnov test assessed the normal distribution of metric data. For non-normally distributed data, the Mann–Whitney U test was applied, while Student's *t*-test was used for normally distributed data. A *p*-value of < 0.05 was considered statistically significant.

#### 2.1.6. Additional Parameters and Considerations

Neuron-Specific Enolase (NSE): NSE levels, a biomarker for neurological damage, were monitored and cross-referenced with imaging findings to validate the presence and extent of brain injury.

Device therapy duration: The duration of ECMO and IABP support was recorded and analyzed to explore any correlation between the length of therapy and the risk of ischemic events. This analysis aimed to identify critical time points where the risk of complications might increase.

Patient outcomes: A detailed survival analysis was conducted, extending beyond the initial 30-day period to capture long-term outcomes associated with brain ischemia in the study cohort. This included functional outcomes and quality-of-life assessments when such data were available.

This comprehensive methodology offers a thorough evaluation of the effects of va-ECMO and IABP on brain ischemia, contributing valuable information on ECMO-associated complications and helping to guide future clinical practices.

It must always be noted that retrospective data collection is not feasible due to the absence of consistent documentation. Therefore, the combined use of mechanical circulatory support systems should be guided by standardized protocols that capture cerebral perfusion pressure, cerebral saturation, and hemodynamic parameters at the time of cerebral events. This will facilitate a better causal explanation of stroke rates whenever possible.

## 3. Results

A total of 146 patients (36 females and 110 males) with an average age of 61.3 years (range: 21–85 years) were included in this study, all of whom underwent va-ECMO therapy. Out of this cohort, 69 patients received simultaneous intra-aortic balloon pump (IABP) treatment. Within this subgroup, 44 patients underwent peripheral cannulation, and 25 patients underwent central cannulation. In contrast, among the 77 patients treated with va-ECMO alone, 66 received peripheral cannulation, while 11 received central cannulation. This detailed distribution of cannulation types provides insight into the management strategies and potential differences in clinical outcomes between the groups.

The baseline characteristics of the two groups (ECMO-only and ECMO + IABP) are presented comprehensively in Table 2. The table includes demographic information, such as age and sex distribution, along with details on underlying conditions that led to the initiation of ECMO therapy. These conditions include cardiogenic shock, myocardial infarction, and cardiac arrest, highlighting the critical status of patients who typically receive this intervention. The table also lists pre-existing risk factors, such as hypertension, diabetes mellitus, and a history of stroke, which are essential for understanding how comorbidities might influence clinical outcomes, complication rates, and overall survival in ECMO therapy.

Additionally, the table provides information on the duration of device therapy for each patient. These data enable an analysis of the relationship between ECMO duration and clinical complications or mortality. By examining the duration of therapy, the study aims to identify any patterns that might suggest optimal or critical periods for intervention or adjustment in patient management. This approach offers nuanced insights into balancing life-saving therapy by minimizing risks such as thrombosis, bleeding, and ischemic events.

Incorporating details on cannulation site specifics also enables a more refined analysis of the impact of peripheral versus central cannulation on patient outcomes. This differentiation is particularly important when comparing the ECMO-only group to those receiving additional IABP therapy, as it may highlight variations in perfusion and complication rates based on the cannulation strategy used. Peripheral and central cannulations present distinct challenges and risks, and understanding their implications is essential for optimizing patient management protocols.

Table 2 offers a detailed comparison of the patient groups, including therapy duration and baseline characteristics, which are instrumental in evaluating the efficacy and safety of va-ECMO with or without concomitant IABP. By providing a comprehensive overview of demographics, pre-existing conditions, and procedural details, the table supports a deeper exploration into how these factors influence patient outcomes and may guide future improvements in ECMO management practices.

The distribution of peripheral versus central cannulation was meticulously documented, as the choice of cannulation site can significantly influence perfusion outcomes and the risk of ischemic events in ECMO patients. Differentiating between these two types of cannulation allows for a more nuanced analysis of how each impacts brain perfusion, particularly when additional intra-aortic balloon pump (IABP) therapy is administered. Understanding these distinctions is crucial, as the study aims to evaluate the impact of combined ECMO and IABP therapy on brain ischemia compared to ECMO-only therapy, thereby providing insights that could guide future clinical decisions.

During the study, 23 out of 146 patients (16%) experienced a total of 26 ischemic stroke events, which were identified via CCT during their treatment. Of these, 23 events (88%) were classified as thromboembolic infarctions, highlighting the predominance of this type of ischemic event in the ECMO population. In the ECMO-only group, 11 out of 77 patients (14%) experienced stroke events, while in the ECMO + IABP group, 12 out of 69 patients (17%) had brain infarctions. Additionally, within the ECMO-only group, three patients experienced a second stroke event during the course of their treatment, emphasizing the recurrent risk of cerebrovascular events in this critically ill population.

Among the 26 total stroke events, 3 were identified as hemodynamic infarctions (12%), and notably, all of these cases occurred within the ECMO + IABP group. The occurrence of hemodynamic infarctions solely in this group suggests that the combination of ECMO and IABP therapy might influence the hemodynamic environment in a way that predisposes patients to this type of infarction. However, despite this observation, statistical analysis did not show a significant difference between the ECMO-only and ECMO + IABP groups regarding the overall infarction rate, infarction type, or survival rate. This lack of significant difference indicates that while there may be trends in the data, larger studies or additional parameters may be needed to confirm the influence of combined therapy on stroke outcomes.

Neuron-specific enolase (NSE), a marker of neuronal injury, was found to be elevated in all patients who experienced infarctions. The levels were higher in the ECMO + IABP group (160 µg/dL) compared to the ECMO-only group (120 µg/d, *p* = 0.72).

Further detailed information, including the distribution of stroke events between the groups, the types of strokes identified, and the 30-day survival rate following ECMO explantation, as well as the involvement of vascular territory in cases of infarction, assessed using the ASPECTS and the modified ASPECTS, is presented in Table 3. Often, several vascular territories were affected during one single stroke event. There were no significant differences regarding involvement of vascular territories or parenchymal involvement according to ASPECTS and modified ASPECTS between the two groups. All patients received conservative treatment because the infarction was already demarked on CCT, so no intravenous lysis or thrombectomy were performed in case of large vessel occlusion.

**Figure 2:**
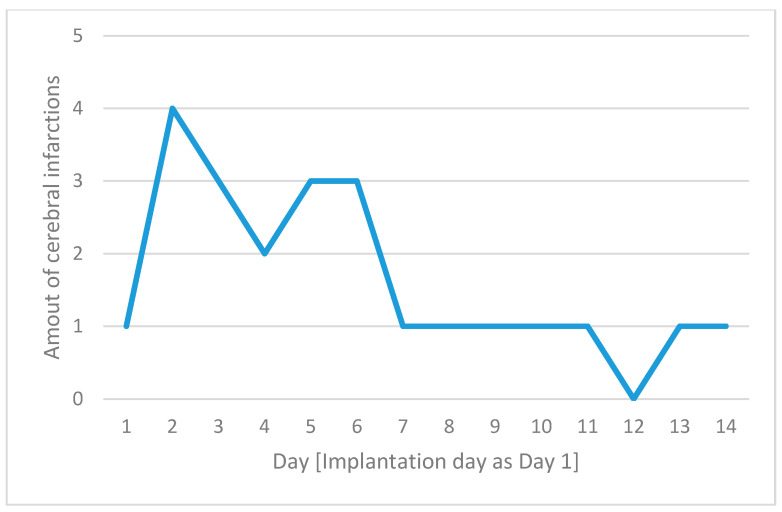
The number of cerebral complications for each day following implantation, with day 1 defined as the day of device implantation on the x-axis and the number of cerebral infarctions on the y-axis.

The amount of infarction per day after implantation is shown in Figure 2.

## 4. Discussion

Our retrospective study emphasizes that ischemic stroke remains a notable complication in va-ECMO therapy, with the majority of events being thromboembolic in nature. These events pose a critical risk that necessitates the further development of strategies for understanding and prevention. The findings suggest that patients receiving combined therapy involving ECMO and intra-aortic balloon pump (IABP) do not experience an increased risk of thromboembolic brain infarctions compared to those treated with ECMO alone. Furthermore, the addition of IABP therapy does not appear to mitigate the risk of hemodynamic infarctions in these patients.

The survival rate in our study’s cohort was notably lower than the survival rates reported by the Extracorporeal Life Support Organization (ELSO) in its 2022 and 2014 data, which indicated survival rates of 59% and 40%, respectively, for va-ECMO patients [14]. Several factors may explain this discrepancy. First, our inclusion criteria were specific, excluding ECMO patients who did not undergo CCT. This selection could have inadvertently omitted patients with potentially better outcomes, thereby skewing our survival data toward a worse outcome. Additionally, a higher proportion of patients in our ECMO-only group presented with cardiopulmonary resuscitation (CPR), accounting for 68% of this group. In contrast, the combination of ECMO with an IABP may have been used less frequently in patients with less severe illnesses, potentially contributing to lower resuscitation rates in this group. Furthermore, research has demonstrated that ECMO is often used more frequently in patients with severe cardiogenic shock, increasing the need for resuscitation, while IABP is more commonly employed in patients with less severe conditions [15]. Differing therapeutic practices may have contributed to variations in outcomes and resuscitation rates [16]. These potential explanations are likely attributable to the retrospective data collection process and the inconsistent documentation of patient characteristics and treatment strategies, leading to biases and random discrepancies. This high resuscitation rate also may reflect the severity of illness in our patients, as our center specializes in the care of highly critical cases, which can negatively impact survival outcomes [17]. This level of severity could also be a contributing factor, as critically ill patients generally have poorer prognoses, regardless of therapeutic interventions, weakening the comparability of the two groups.

The absence of a significant difference in 30-day survival between the ECMO-only and ECMO + IABP groups is consistent with previous findings, such as those of Lin et al., who reported nearly identical two-week mortality rates of 48.5% in the IABP group versus 47% in the ECMO-alone group [11]. Gass et al. also found an in-hospital survival rate of 57.8% for va-ECMO and IABP patients (*p* = 0.053) [18]. Collectively, these data suggest that using IABP does not provide a survival advantage. This lack of clear benefit should be carefully weighed against the risks associated with IABP use in clinical decision-making.

The brain infarction rate in our study was 16% overall, with rates of 14% in the ECMO-only group and 17% in the ECMO + IABP group. These rates are in line with the wide range reported in the literature, where the brain infarction incidence in va-ECMO therapy varies from 3% to 33% [7,19,20]. This variability may stem from differences in diagnostic criteria, patient populations, and ECMO management strategies across studies. It is crucial to distinguish between different ECMO types when discussing stroke risk; for instance, venovenous ECMO involves the lungs, which act as a filter for emboli, offering a degree of protection to the brain. The protective role of the lungs in venovenous ECMO highlights the importance of carefully selecting the appropriate ECMO modality based on the patient’s risk profile. Moreover, some studies diagnose ischemic events based solely on written radiological reports, while our study utilized second image readings, which may have enhanced our detection accuracy [7,21,22]. To our knowledge, no previous studies have specifically reported on the stroke incidence and infarction types in patients undergoing combined va-ECMO and IABP therapy, making our findings an important contribution to the field.

Thromboembolic events were identified as the predominant cause of infarctions in our cohort. These emboli may originate from multiple sources, including the ECMO circuit itself, the pulmonary veins, or the heart. In the case of cardiac thrombus formation, slow blood flow or reduced cardiac wall motion could lead to the development of thrombus, which can eventually detach and enter cerebral circulation [23]. In scenarios involving peripheral cannulation, thrombotic material might bypass the IABP during the diastole and enter the aortic arch. On the other hand, thrombi from the left ventricle or central ECMO cannula could reach the supra-aortic vessels through the antegrade flow [12,23]. The IABP’s role in enhancing perfusion to the supra-aortic branches might alter the watershed location, thereby raising the risk of thromboembolic events from the ECMO system especially in cases of peripheral cannulation [24,25,26,27]. Despite our inability to demonstrate statistically significant differences in thromboembolic infarctions between groups, the higher CPR rate in the ECMO-only group may have skewed these results, as CPR itself is a recognized risk factor for thromboembolism. Similarly, more extensive neuronal damage was detected in patients receiving the combined therapy. However, the statistical analysis of NSE levels between the two groups did not demonstrate a significant difference, indicating that while there is an observable difference in NSE levels, it may not be clinically meaningful, or a larger sample size may be required to determine its significance.

Embolism and hypoperfusion seem to play synergetic roles [28,29]. In cases of lower levels of perfusion in delivering vessels, a thrombus that would typically pass the capillary system under normal hemodynamic circumstances is more likely to obstruct a terminal vessel and cause infarction [30]. Hemodynamic infarction was less common, with only three cases observed, all occurring within the ECMO + IABP group. This low incidence suggests that hemodynamic infarction is relatively rare in ECMO therapy, and the addition of IABP did not appear to provide any protective effect against these events. The absence of ECMO-related hypoxic infarctions in our cohort indicates that this type of infarction may be of limited relevance in the context of ECMO therapy. However, other authors have reported hypoxemic infarctions [31] and our data show a trend of increased risk of hemodynamic infarction in the ECMO + IABP group, which is perhaps not statistically significant due to the sample size. Our data further indicate that the risk of thromboembolic events is highest within the first 48 h following ECMO implantation, emphasizing the importance of vigilant monitoring during this critical period to promptly identify and manage these events. The early occurrence of strokes in patients undergoing ECMO can be attributed to a complex interplay of mechanisms and patient-specific factors. The first days of ECMO support are critical due to the combination of the patient’s underlying critical condition and the physiological changes induced by the ECMO system. Patients requiring ECMO often have severe statuses that predispose them to thrombotic or embolic events. These pre-existing conditions, combined with the inherent risks of ECMO, significantly increase the likelihood of stroke early in the course of support, particularly before anticoagulation therapy is fully optimized [32].

The retrospective design of our study presented limitations, especially in assessing all relevant comorbidities or different coagulation protocols that could contribute to the risk of intracranial infarction. While CCT is an effective tool for detecting brain infarction, magnetic resonance imaging (MRI) could have offered superior resolution and potentially higher detection rates. Conventional MRI is not feasible in this arrangement, and so portable bedside MRI has been employed in a few studies [33]. The absence of a true control group limits the comparability of either intervention. Differences in CPR rates, cannulation sites, and the timing of CCT acquisition between the two groups might also have introduced biases that influenced our results. The absence of multivariate modeling is a significant limitation, as it hinders the ability to account for potential confounders such as age, gender, and comorbidities. For outcomes like ischemic stroke, logistic regression, or others, multivariable techniques should be utilized to adjust for these confounders and enhance the validity of the findings. Additionally, the limited sample size and retrospective nature of the study further restrict its reproducibility and external validity, compromising the generalizability of the results and undermining confidence in their clinical implications. The study’s 5-year duration may have also confounded the results, as advancements in ECMO or IABP therapy during this period could have led to varying outcomes. Standardizing these factors in future studies could provide a more accurate understanding of the risks associated with ECMO and IABP therapy, ensuring more robust and generalizable findings. Low statistical power may reduce the likelihood of identifying significant associations or differences, especially when small sample sizes are involved. Due to these limitations, our study had the potential for Type II errors, where true effects may be present but are not detected due to an insufficient sample size or variability in patient characteristics.

The primary goal of our study was to compare ECMO-only therapy with combined ECMO and IABP therapy, offering initial insights into the types of brain infarctions in va-ECMO patients. In summary, this study provides a detailed account of the stroke events in ECMO therapy, emphasizing the predominance of thromboembolic events and the complexity of managing such complications in patients receiving combined ECMO and IABP therapy. Our findings highlight that brain infarctions, which are predominantly thromboembolic, remain a critical and frequent complication in ECMO therapy. The addition of IABP does not appear to reduce the risk of ischemic stroke, including hemodynamic strokes, which were infrequent in our cohort. Hypoxemic infarctions were also rare, regardless of the cannulation site, suggesting that they are less relevant in this context. To deepen the understanding of the hemodynamic mechanisms and the potential sources of thromboembolic events in ECMO and IABP therapy, further studies should include prospective data collection, including echocardiographic evaluations, to assess cardiac and aortic flow dynamics more accurately.

In conclusion, the decision to use additional IABP therapy in ECMO patients should be guided by considerations other than the risk of brain ischemia, as our retrospective study demonstrates that it does not significantly alter the likelihood of ischemic stroke. It is essential to interpret these findings with caution, considering the study’s design, sample size, and limitations specific to the population. Given the declining use of IABP in favor of more frequently deployed devices such as the Impella, further prospective studies are essential to evaluate the impact of these newer mechanical circulatory support strategies on cerebrovascular outcomes in ECMO patients. The focus in future studies must be on multivariate modeling and standardized protocols to ensure transparency and improve interpretability.

## Figures and Tables

**Table 1:** Four different CT models employed in this study.

Model	Siemens Definition AS Plus	Siemens Definition AS with Sliding Gantry	Siemens Definition Flash	Siemens Emotion
Slices	128	64	2 × 128	16
Reconstruction slice thickness	2 mm	2 mm	2 mm	3 mm infratentorial/ 5 mm supratentorial
Tube voltage	100 kV	100 kV	120 kV	130 kV
Field of view	213 mm	220 mm	220 mm	220 mm
Automatic dose exposure control (Siemens care dose 4 D)	Yes	Yes	Yes	No

**Table 2:** Baseline characteristics, duration of device therapy, cannulation site, and indication for ECMO-therapy.

	ECMO Only (*n* = 77)	ECMO + IABP (*n* = 69)	*p*-Values
**Age (years)**	58.5 (±13.0)	64.5 (±13.0)	0.98
**Sex**	21 females, 27%	15 females, 22%	0.60
**Duration of device therapy in days mean (±SD)**	7.1 (±5.9)	ECMO: 9.7 (±6.1) IABP: 8.2 (±4.6) Simultaneous: 6.8 (±4.2)	0.60
**Duration of ECMO without IABP in days**	N/A	2.9 (±4.8)	N/A
**Duration of IABP without ECMO (days)**	N/A	1.3 (±1.9)	N/A
**Cannulation site**	66 peripheral (85.7%) 11 central (14.3%)	44 peripheral (60.9%) 25 central (39.1%)	N/A
**Indication**	Coronary artery disease and myocardial infarction: 44% Cardiac decompensation: 9% Cardiac arrhythmia: 4% Heart transplantation: 3% Aortic dissection: 3% Other: 7% Unknown: 30%	Coronary artery disease and myocardial infarction: 47% Artificial heart valve: 22% Cardiac decompensation: 17% Aortic dissection: 7% Heart transplantation: 1% Cardiac tamponade: 1% Unknown: 5%	N/A

SD = standard deviation, IABP = intra-aortic balloon pump, ECMO = extracorporeal membrane oxygenation.

**Table 3:** Stroke rate, type of infarction, localization, ASPECTS, and modified ASPECTS, and survival rate in ECMO-only- and ECMO + IABP-therapy.

	ECMO Only (N = 77)	ECMO + IABP (N = 69)	*p*-Values
**Patients with cerebral infarctions**	11	12	0.61
**Total no. of events with cerebral infarction**	14	12	0.44
**Hemodynamic cerebral infarctions (no. of events)**	0	3	0.1
**Thromboembolic cerebral infarctions (no. of events)**	14	9	0.61
**Vascular territories involved in thromboembolic infarctions**			
ACA	3	1	
ACM	10	72	
ACP	8	6	
SCA	3	3	
AICA	3	2	
PICA	4	4	
BA	1	1	
**Hypoxic cerebral infarctions (no. of events)**	0	0	N/A
**ASPECTS right MCA territory (median)**	9	10	N/A
**ASPECTS left MCA territory (median)**	9	10	N/A
**Modified ASPECTS right (median)**	17	17	N/A
**Modified ASPECTS left (median)**	17	18	N/A
**Survival rate within 30 days of treatment (%)**	29	32	0.66
**Cause of death during ECMO therapy**	Multi organ dysfunction syndrome (49%) Cardiac failure (16%) Bleeding (5%) Neurological complication (11%) Other (19%)	Multi organ dysfunction syndrome (57%) Cardiac failure (19%) Bleeding (4%) Neurological complication (8%) Other (12%)	
**CPR-rate (%)**	68	36	<0.01

SD = standard deviation, IABP= intra-aortic balloon pump, ECMO = extracorporeal membrane oxygenation, CPR = cardiopulmonary resuscitation, ASPECTS = Alberta Stroke Program Early CT Score, ACA = anterior cerebral artery, ACM = middle cerebral artery, ACP = posterior cerebral artery, SCA = superior cerebellar artery, AICA = anterior inferior cerebellar artery, PICA = posterior inferior cerebellar artery, BA = basilar artery.

## Data Availability

The data presented in this study are available on reasonable request from the corresponding author due to patient privacy.

## Abbreviations

CCT	cranial computed tomography
CPR	cardiopulmonary resuscitation
IABP	intra-aortic balloon pump
va-ECMO	veno-arterial extracorporeal membrane oxygenation
ASPECT	Alberta Stroke Program Early CT Score
